# Retraction Note: Tumour-originated exosomal miR-155 triggers cancer-associated cachexia to promote tumour progression

**DOI:** 10.1186/s12943-023-01728-8

**Published:** 2023-02-01

**Authors:** Qi Wu, Si Sun, Zhiyu Li, Qian Yang, Bei Li, Shan Zhu, Lijun Wang, Juan Wu, Jingping Yuan, Changhua Yang, Juanjuan Li, Shengrong Sun

**Affiliations:** 1 Department of Breast and Thyroid Surgery, Renmin Hospital of Wuhan University, Wuhan, 238 Ziyang Road, Wuhan, 430060 Hubei People’s Republic of China; 2 Department of Clinical Laboratory, Renmin Hospital of Wuhan University, Wuhan, Hubei People’s Republic of China; 3 Department of Pathology, Renmin Hospital of Wuhan University, Wuhan, Hubei People’s Republic of China; 4 Department of Pathology and Pathophysiology, Wuhan University School of Basic Medical Sciences, Wuhan, 430060 Hubei Province People’s Republic of China

**Retraction note: Mol Cancer 17, 155 (2018)**

**https://doi.org/10.1186/s12943-018-0899-5**

The Editor-in-Chief has retracted this article. After publication, concerns were raised regarding high similarity with the authors' other article that was under consideration within a similar time-frame [1]. Specifically:

- Fig. 1b Exosomes/CytoD images appear to overlap with Fig. 6c Exosomes/PRO in [1].
- Fig. 1f UCP1 appear to be duplicated in Fig. 5d UCP1 in [1].
- Fig. 2a panel 2 appear to be duplicated in Fig. 1c panel 2 in [1].

In addition, the western blots in Fig. 2C UCP3 (C2C12) and Fig. 3H UCP1 (Adipocytes) appear to originate from the same gel. The authors have provided the raw data to address these concerns; however, these data contained a number of further discrepancies. The Editor-in-Chief therefore no longer has confidence in the presented data.

Qi Wu agrees to this retraction. None of the other authors have responded to any correspondence from the publisher about this retraction.
